# High-molecular-weight adiponectin levels in healthy, community-dwelling, elderly Japanese volunteers: a 5-year prospective observational study

**DOI:** 10.1007/s40520-017-0840-6

**Published:** 2017-10-19

**Authors:** Hiromasa Otsuka, Mitsuru Yanai, Hiroki Kobayashi, Akira Haketa, Motohiko Hara, Kaoru Sugama, Kimitoshi Kato, Masayoshi Soma

**Affiliations:** 1 Division of General Medicine, Department of Internal Medicine, Nihon University School of Medicine, Oyaguchikamimachi 30-1, Itabashi-ku, Tokyo, Japan; 2 Division of Nephrology, Hypertension and Endocrinology, Department of Internal Medicine, Nihon University School of Medicine, Tokyo, Japan; 3 Department of Nursing, School of Health and Social Services, Saitama Prefectural University, Tokyo, Japan; 4 Division of Cell Regeneration and Transplantation, Department of Functional Morphology, Nihon University School of Medicine, Tokyo, Japan

**Keywords:** Body weight, High-molecular-weight adiponectin, Prospective study, Muscle strength

## Abstract

**Background:**

Serum adiponectin levels are associated with frailty and cardiovascular diseases. Longitudinal changes in adiponectin levels might enhance our understanding of age-related conditions and diseases.

**Aims:**

This prospective observational study aimed to: (1) elucidate age-related changes in high-molecular-weight (HMW) adiponectin levels; and (2) identify variables predictive of elevated HMW adiponectin levels and the association with well-known adiponectin single-nucleotide polymorphisms (SNPs) in healthy, elderly Japanese participants.

**Methods:**

Healthy elderly volunteers (*n* = 196; 55 men and 141 women; median age 72.0 years; range 69.0–75.0 years) underwent anthropometric and physical function measurements, as well as laboratory tests at baseline and the 5-year follow-up.

**Results:**

HMW adiponectin levels were significantly higher in women than in men (8.4, 5.3–11.9 vs. 5.7, 3.1–9.0 μg/mL; *p* < 0.001) at baseline and decreased significantly at follow-up in women (7.7, 4.8–11.2 μg/mL; *p* < 0.001), but not in men. In the multiple regression analysis, high-density lipoprotein cholesterol levels and body weight were independent predictors of HMW adiponectin levels. The rate of change in HMW adiponectin levels was inversely correlated with the rates of change in body weight, body mass index, and knee leg extension strengths, and positively correlated with rates of change in high-density lipoprotein cholesterol and one-leg standing time. There were no significant differences in HMW adiponectin levels among SNPs.

**Discussion:**

Decreasing HMW adiponectin levels might lead to an increased risk of cardiovascular diseases in elderly women.

**Conclusion:**

HMW adiponectin levels significantly decreased over a 5-year period in community-dwelling elderly Japanese women.

## Introduction

Adiponectin is an adipose tissue-derived specific protein encoded by the adiponectin gene (ADIPOQ) located on chromosome 3q27 in a region identified as a susceptibility locus for metabolic syndrome [1]. Serum levels of adiponectin have been associated with glucose intolerance, diabetes mellitus, and cardiovascular diseases (CVD) [2, 3]. Common single-nucleotide polymorphisms (SNPs) at the ADIPOQ locus (SNP + 45T/G and SNP + 276G/T) have also been associated with low adiponectin levels, insulin resistance, and diabetes mellitus in a Japanese population [4].

Several studies have demonstrated that serum total adiponectin levels increase with age [5, 6]. Moreover, cross-sectional studies have reported higher circulating total adiponectin levels in older adults compared to those in younger adults [7]. Conversely, in a recent analysis of older Korean adults, age was associated with total adiponectin levels in males but not females [8], suggesting the sex-associated differences. According to these reports, age and sex should be considered in the evaluation of plasma adiponectin levels. Although serum adiponectin levels are associated with the incidence of physical disability, CVD, and mortality in the elderly [3, 9], the association between serum adiponectin levels and healthy elderly individuals is yet to be reported. Therefore, longitudinal changes in adiponectin levels may be useful to understand healthy aging and age-related diseases and/or conditions. However, no studies to date have prospectively evaluated the changes in adiponectin levels and the related genetic impact over time in a healthy elderly population.

To this end, we conducted a prospective observational study to assess whether high-molecular-weight (HMW) adiponectin levels changed over a period of 5 years in elderly Japanese individuals. We identified variables predictive of elevated HMW adiponectin levels and investigated the association between plasma HMW adiponectin levels and SNPs in ADIPOQ.

## Methods

### Study design

The Ogano study is an ongoing prospective observational study initiated in 2004 as an investigation of longevity in healthy adults in Ogano-machi, a town of approximately 12,000 residents located in the Saitama Prefecture in Japan. The recruitment of healthy volunteers was conducted using pamphlets annually disseminated throughout the city. Annual participant evaluations were conducted at the Ogano assembly hall and included standardized questionnaires, anthropometric measurements, as well as physical function and laboratory tests, including the collection of blood samples for the measurement of HMW adiponectin levels. We conducted health assessment interviews for all participants. Participants who had mobility limitations, severe health conditions, or injuries within 3 months before the study and those with motor dysfunctions, mental disorders, or cognitive impairments, were excluded. By 2008, 718 residents (men *n* = 244; women *n* = 474) had participated in the original study. We sent postal mail to recruit participants for whom 5 years had passed since the first study visit to perform follow-up evaluations. By 2013, 222 of the original 718 participants (30.9%) had been followed up for 5 years. We conducted the same health assessments at the follow-up. We used the participants who were followed up to evaluate changes in HMW adiponectin levels in the elderly. The participants who were followed up were excluded because their age was < 65 years, and 26 participants were excluded for being < 65 years old. Finally, 196 participants (men *n* = 55; women *n* = 141) were enrolled and analyzed in the current study.

### Data collection

The participants were asked to answer a standard questionnaire regarding their current smoking status, regular exercise habits, medical history (treatment for hypertension and diabetes mellitus), and family history. Questions were asked face-to-face by trained interviewers to ensure the quality and accuracy of the answers. Height and body weight measurements were conducted with the participants wearing light clothing and no shoes. Body mass index (BMI) was calculated using the following formula: weight (kg)/height (m^2^). Waist circumference was defined as the smallest girth midway between the lowest rib and the iliac crest at the end of normal expiration. To stabilize blood pressure in a seated position, the participants rested for 5 min before their blood pressure was measured twice from the right arm with a standard mercury sphygmomanometer, and the mean of these two measurements was recorded as the blood pressure value.

#### Measures of physical function

Physical function measurements included hand grip strength, knee extension strength, and one-leg standing time. Participants were guided by trained instructors before each examination to learn the procedures. Hand grip strength was measured for each hand with a dynamometer adjusted to fit the participant’s hand size, and the test was performed in a standing position. Knee extension strength was measured in two maximum knee extension efforts against a bilaterally positioned force sensor while the participant was seated. The one-leg standing time was measured using a stopwatch, for both legs and with eyes open. These measurements were performed once, and the means of each recorded value were used for the analyses of hand grip strength and knee extension strength. The better time was used for the analyses of one-leg standing time, unless the participants performed the test incorrectly.

#### HMW adiponectin, biochemical marker, and bone mineral density measurements

- Blood samples were obtained from all participants from the cubital vein while non-fasting and at rest and stored at −80 °C until the analysis. Measured laboratory parameters included serum HMW adiponectin, serum total cholesterol levels, and serum high-density lipoprotein (HDL) cholesterol levels. HMW adiponectin levels were determined via a chemiluminescent enzyme immunoassay using a Lumipulse^®^ ƒ analyzer (Fujirebio, Tokyo, Japan). Intra-assay and inter-assay coefficients of variation were 5.2–6.9 and 2.8–4.5%, respectively [10]. The total cholesterol was measured using a cholesterol ester hydrolase–cholesterol dehydrogenase-ultraviolet method [11]. The direct measurements of HDL cholesterol [12] was conducted at a central laboratory (SRL Inc., Tokyo, Japan). Non-HDL cholesterol levels were expressed as the total cholesterol levels minus HDL cholesterol levels. Bone mineral density was determined at the calcaneus with a pulse-echo ultrasound method using a bone density measuring instrument (CM-100, Canon Lifecare Solutions Inc., Osaka, Japan).

### Single-nucleotide polymorphism analysis

Genomic DNA was extracted from peripheral blood leukocytes via the phenol–chloroform method [13]. Genotyping was performed using the TaqMan^®^ SNP genotyping assay (Applied Biosystems, Foster City, CA, USA) [14]. Three ADIPOQ SNPs, namely + 276G/T (rs1501299) and + 349A/G (rs2241767) in an intron and + 45T/G (rs2241766) in an exon, were selected after careful review of the literature, and were included in the current study based on their significant association with serum adiponectin levels [4].

The TaqMan^®^ SNP genotyping assays used were C_7497299_10 (rs1501299), C_26426076_10 (rs2241767), and C_26426077_10 (rs2241766). PCR amplification was performed with a 5 μL final reaction volume containing 2.5 μL TaqMan^®^ universal master mix, 2 ng DNA, 2.375 μL ultrapure water, 0.079 μL Tris–EDTA buffer (1 ×), and 0.046 μL TaqMan^®^ SNP genotyping assay mix (40 ×) containing the primers (final concentration 331.2 nmol/L) and probes (final concentration 73.6 nmol/L). Thermal cycling was performed using the GeneAmp 7700 system (Applied Biosystems, Foster City, CA, USA) under the following conditions: 50 cycles of 50 °C for 2 min and 95 °C for 10 min and 50 cycles of 95 °C for 15 s and 60 °C for 1 min. Each 96-well plate contained 80 DNA samples of an unknown genotype and four reaction mixtures containing reagents without DNA as controls, which were a necessary part of the 7700-signal processing system, as outlined in the TaqMan^®^ Allelic Discrimination Getting Started Guide (Applied Biosystems, Foster City, CA, USA). The plates were read on an ABI Prism 7700 sequence detection system in endpoint analysis mode (version 1.6.3; Applied Biosystems, Foster City, CA, USA). Genotypes were determined visually based on the dye component fluorescent emission data depicted on *X*–*Y* scatter plots by the sequence detection system software. Genotypes were also determined automatically by the software’s signal processing algorithms. The results of each scoring method were saved in separate output files for subsequent comparisons [15].

### Statistical analysis

All statistical analyses were performed using SPSS version 22.0 for Windows (SPSS, Japan Inc., Tokyo, Japan). Continuous variables were expressed as the mean ± standard deviation or median (25th–75th percentile), for normal and non-normal distributions, respectively. Categorical data were expressed as percentages, and *χ* ^2^ or Fisher’s exact tests were used to compare men and women. The distribution of variables was tested using the Kolmogorov–Smirnov test, and differences in the continuous variables between the first and 5-year follow-up visits were analyzed using paired Student’s *t* tests or paired Wilcoxon signed-rank tests. Baseline values and rates of change for all variables were assessed to determine their association with serum HMW adiponectin levels using the univariate correlation test and Spearman’s rank correlation coefficient (*r*_s_). Moreover, a multiple regression analysis was performed using the stepwise method to determine independent predictors of serum HMW adiponectin levels. HMW adiponectin levels were natural logarithm transformation. We adjusted for age, sex, body weight, waist circumference, hand grip strength, knee extension strength, one-leg standing time, bone mineral density, HDL cholesterol, and history of diabetes mellitus. We also analyzed the rate of change in the HMW adiponectin levels using a multiple regression analysis. Moreover, all analyses were performed separately for each sex, and the differences between men and women were analyzed using a Mann–Whitney *U* test. Differences in the serum HMW adiponectin levels among the ADIPOQ SNPs were evaluated via a Kruskal–Wallis H test. The consistency of genotype distribution with the Hardy–Weinberg equilibrium was calculated using a *χ* ^2^ test. All tests were two-tailed, and *p* values of less than 0.05 were considered statistically significant.

## Results

Table 1 presents the baseline and follow-up characteristics of all participants, as well as those for men and women separately. The proportion of current smokers and regular exerciser comprised 10 and 15.9% of the participants, respectively. Among all participants, the median HMW adiponectin and bone mineral density values, as well as all measured physical functions were significantly decreased at the 5-year follow-up. Height, body weight, and BMI were also significantly decreased for all participants. In contrast, there was no significant change in the mean waist circumference and non-HDL cholesterol levels. HMW adiponectin levels at baseline were inversely correlated with height (*r*_s_ = − 0.291; *p* < 0.001), body weight (*r*_s_ = − 0.338; *p* < 0.001), BMI (*r*_s_ = − 0.190; *p* = 0.008), hand grip strength (*r*_s_ = − 0.234; *p* = 0.001), and knee extension strength (*r*_s_ = − 0.177; *p* = 0.014). Conversely, HDL cholesterol levels were positively correlated with baseline HMW adiponectin levels (*r*_s_ = 0.400; *p* < 0.001).

**Table 1:** Baseline and 5-year follow-up characteristics of the study subjects

Variables	All				Men				Women			
	*n*	Baseline	Follow-up	*p* value	*n*	Baseline	Follow-up	*p* value	*n*	Baseline	Follow-up	*p* value
Age (years)	196	72.0 (69.0–75.0)	77.1 (74.0–80.9)	< 0.001	55	73.0 (70.0–77.0)	78.0 (75.0–82.0)	< 0.001	141	72.0 (68.0–74.5)	77.0 (74.0–80.0)	< 0.001
Height (cm)	193	150.7 (146.1–157.4)	149.7 (144.8–156.1)	< 0.001	54	162.3 (157.6–166.5)	160.5 (156.9–165.3)	< 0.001	139	148..5 (144.4–152.0)^*b*^	147.3 (142.9–150.9)	< 0.001
Body weight (kg)	194	53.1 (48.2–59.0)	51.2 (46.4–57.8)	< 0.001	54	61.2 (55.5–67.1)	60.1 (53.9–64.1)	0.001	140	51.0 (46.9–55.4)^*b*^	49.6 (44.9–54.2)	< 0.001
BMI (kg/cm^2^)	193	23.2 ± 2.8	22.9 ± 2.8	0.001	54	23.1 ± 2.6	22.8 ± 2.7	0.085	139	23.3 ± 2.9	22.9 ± 2.9	0.007
Waist circumference (cm)	99	83.0 ± 8.7	84.7 ± 9.2	0.800	27	86.5 ± 8.3	85.8 ± 8.1	0.344	72	81.7 ± 8.5^*a*^	82.1 ± 9.1	0.691
Systolic blood pressure (mmHg)	192	142 (130–154)	142 (130–155)	0.859	53	142 (131–153)	146 (132–159)	0.580	139	142 (130–154)	140 (130–155)	0.610
Diastolic blood pressure (mmHg)	193	78 (72–86)	76 (69–83)	0.008	53	78 (72–86)	75(70–80)	0.056	140	78 (71–88)	78 (68–84)	0.053
Hand grip strength (kg)	192	24.0 (20.1–28.9)	20.6 (17.1–25.4)	< 0.001	53	33.5 (28.9–38.4)	29.5 (23.4–35.0)	< 0.001	139	22.5 (19.1–25.0)^*b*^	19.3 (14.8–22.0)	< 0.001
Knee extension strength (kg)	182	19.2 (15.1–22.9)	16.2 (13.0–19.5)	< 0.001	51	22.4 (17.9–24.6)	18.4 (14.8–22.3)	0.017	131	18.0 (13.9–21.9)^*b*^	15.4 (12.8–18.1)	< 0.001
One leg standing time (s)	187	59.9 (18.9–89.9)	30.5 (9.3–75.0)	< 0.001	51	56.1 (26.0–136.6)	34.0 (10.9–78.0)	0.045	136	60.0 (15.8–82.2)	28.4 (8.9–74.3)	< 0.001
Bone mineral density (g/m^2^)	175	1504 (1491–1523)	1481 (1471–1498)	< 0.001	45	1514 (1495–1531)	1491 (1477–1513)	< 0.001	130	1502 (1491–1516)^*a*^	1479 (1468–1493)	< 0.001
HMW adiponectin (µg/mL)	194	7.7 (4.6–10.9)	7.1 (4.2–10.3)	< 0.001	55	5.7 (3.1–9.0)	5.0 (3.2–8.6)	0.321	141	8.4 (5.3–11.9)^*b*^	7.7 (4.8–11.2)	0.001
Total cholesterol (mg/dL)	92	201 ± 35	197 ± 36	0.186	27	178 ± 30	176 ± 27	0.767	65	211 ± 32^*a*^	206 ± 36	0.173
HDL cholesterol (mg/dL)	104	56 (47–66)	57 (47–66)	0.803	32	49 (44–59)	55 (45–61)	0.880	72	58 (49–70)^*a*^	58 (50–68)	0.699
Non-HDL cholesterol (mg/dL)	92	144 ± 34	139 ± 33	0.131	27	126 ± 29	122 ± 25	0.318	65	152 ± 33^*a*^	147 ± 33	0.238
Hypertension being treated, *n* (%)	196	87/196 (44.4)				25/55 (40.0)				62/141 (44.0)		
Diabetes mellitus being treated, *n* (%)	196	13/196 (6.6)				2/55 (3.6)				11/141 (7.8)		
Coronary artery disease, *n* (%)	196	27/196 (13.8)				11/55 (20.0)				16/141 (11.3)		
Cerebral vascular disease, *n* (%)	196	14/196 (7.1)				8/55 (14.5)				6/141 (4.3)^*a*^		
Cancer, *n* (%)	196	14/196 (7.1)				7/55 (12.7)				7/141 (5.0)		

Continuous data are mean ± standard deviation or median (25th–75th percentile). Categorical data are percentage

*BMI* body mass index, *HMW* high-molecular-weight, *HDL* high-density lipoprotein

^a^ *p* < 0.05 men vs. women at baseline

^b^ *p* < 0.001 men vs. women at baseline

At baseline, the men and women significantly differed regarding several parameters, including height, body weight, waist circumference, hand grip strength, knee extension strength, bone mineral density, HMW adiponectin levels, total cholesterol levels, HDL cholesterol levels, non-HDL cholesterol levels, and cerebral vascular disease. At the 5-year follow-up, serum HMW adiponectin levels were significantly decreased in the women but not in men. Both men and women exhibited decreased height and body weight, and similar decreases in hand grip and knee extension strength were observed for both sexes at the 5-year follow-up. In contrast, the waist circumference and non-HDL cholesterol levels did not change. Furthermore, the rate of change in the HMW adiponectin levels in women was significantly inversely correlated with the rates of change in body weight (*r*_s_ = − 0.334; *p* < 0.001), BMI (*r*_s_ = − 0.295; *p* < 0.001), and knee extension strength (*r*_s_ = − 0.187; *p* = 0.032). Conversely, the rates of change in HMW adiponectin levels were positively correlated with the rates of change for the one-leg standing time and HDL cholesterol (*r*_s_ = 0.231; *p* < 0.007 and *r*_s_ = 0.273; *p* = 0.021, respectively). None of the examined variables were significantly correlated with the rate of change in the HMW adiponectin levels in men.

The multiple regression analysis revealed HDL cholesterol and body weight as independent predictors of serum HMW adiponectin levels (Table 2). Additionally, the rate of change in HMW adiponectin levels was significantly inversely correlated with rates of change in body weight (*r*_s_ = − 0.292; *p* < 0.001), BMI (*r*_s_ = − 0.267; *p* < 0.001) and knee extension strength (*r*_s_ = − 0.183; *p* = 0.014). In contrast, the rate of change in HMW adiponectin levels was significantly positively correlated with the rates of change for the one-leg standing time (*r*_s_ = 0.220; *p* = 0.003) and HDL cholesterol (*r*_s_ = 0.205; *p* = 0.037). In the multiple linear regression analysis, rates of change in body weight and HDL cholesterol were independent predictors of the rate of change in HMW adiponectin levels.

**Table 2:** Multiple regression analysis for log-transformed high-molecular-weight adiponectin at baseline (*n* = 196)

Significant variable	*β*	SE	*p* value
Constant	2.237	0.626	0.001
Body weight (kg)	− 0.017	0.008	0.037
HDL cholesterol (mg/dL)	0.011	0.005	0.044

Independent variables = age, sex, body weight, waist circumference, hand grip strength, knee extension strength, one-leg standing time, bone mineral density, HDL cholesterol, and medical history of diabetes mellitus

*HDL* high-density lipoprotein

Table 3 presents the rates of change in body weight and HDL cholesterol as independent predictors of the rate of change for HMW adiponectin levels in women. In contrast, no independent variables were found to be predictive of the rate of change in HMW adiponectin levels in men.

**Table 3:** Multiple regression analysis for the rate of change in high-molecular-weight adiponectin in women (*n* = 141)

Significant variables	*β*	SE	*p* value
Constant	− 0.187	0.076	0.016
Rate of change in HDL cholesterol (mg/dL/year)	0.154	0.033	< 0.001
Rate of change in body weight (kg/year)	− 0.225	0.111	0.047

Independent variables = rates of change in body weight, knee extension strength, HDL cholesterol, and medical history of diabetes mellitus

*HDL* high-density lipoprotein

Table 4 displays the difference in HMW adiponectin levels among SNPs. At baseline, no significant differences were noted in the SNPs. Men and women were also analyzed separately, and the dominant and recessive model, as well as the allelic models were evaluated. However, the results revealed no significant association in any of the SNPs at baseline (data not shown). At the 5-year follow-up, HMW adiponectin levels among SNPs (Table 4) did not differ significantly, and the same findings were obtained in the dominant, recessive, and allelic models (data not shown).

**Table 4:** High-molecular weight adiponectin levels at baseline and 5-year follow-up for adiponectin single-nucleotide polymorphisms (SNPs)

Variants	*n* = 196	Baseline	*p* value	Follow-up	*p* value	Minor allele	MAF	HWE (*p* value)
+ 276G/T (rs1501299)						T	0.186	0.504
GG	123	7.0 (3.9–10.7)	0.115	6.2 (3.8–10.3)	0.396			
GT	61	8.1 (5.5–11.2)		7.5 (5.4–10.1)				
TT	12	9.2 (7.2–12.0)		8.1 (4.7–11.6)				
+ 349A/G (rs2241767)						G	0.319	0.535
AA	71	7.2 (4.3–10.3)	0.443	6.6 (4.0–8.9)	0.195			
AG	100	8.3 (4.9–11.2)		7.5 (4.3–11.0)				
GG	25	6.7 (4.2–12.0)		7.3 (4.1–11.9)				
+ 45T/G (rs2241766)						T	0.314	0.782
TT	26	7.0 (4.5–12.0)	0.574	7.7 (4.2–11.9)	0.181			
TG	97	8.1 (4.7–11.6)		7.4 (4.3–11.2)				
GG	73	7.6 (4.5–10.4)		6.6 (4.0–8.9)				

Data are median (25th–75th percentile)

*MAF* minor allele frequency, *HWE* Hardy–Weinberg equilibrium

## Discussion

In this study, we determined that the HMW adiponectin levels were significantly decreased in women but not men during the 5-year follow-up period, and body weight and HDL cholesterol as predictors of HMW adiponectin levels. Previous studies have also reported that body weight and HDL cholesterol were correlated with adiponectin levels, consistent with our results [5, 16]. However, longitudinal HMW adiponectin levels were significantly lower, despite the decreased body weight and inverse correlation between body weight and change in HMW adiponectin levels. The proportion of body weight reduction observed in this study was low compared to that observed in other studies. Therefore, a reduction in body weight might have had a weak effect of the HMW adiponectin levels in the current study. Since body weight reduction in healthy elderly individuals is low, it might be unrelated to longitudinal changes in adiponectin levels.

The proportion of HMW adiponectin to total adiponectin varies by age [17] and sex [18], with women exhibiting higher proportions of HMW adiponectin than that in men, as found in the present study. A reduction in HMW adiponectin levels was observed in women but not men at the 5-year follow-up in the current study. The failure of the decrease in HMW adiponectin levels to reach statistical significance among men might be partially due to the small number of male participants included in the study.

Waist circumference was found to be an independent predictor of visceral fat [19]. In addition, a decrease in muscle mass was shown to be associated with a decrease in muscle strength [20]. In the present study, waist circumference was unchanged at the follow-up; whereas muscle strength had decreased. These results might reflect an increase in visceral or intramuscular fat compared with the levels at baseline. Moreover, HMW adiponectin levels were recently shown to be inversely correlated with visceral fat mass [21]. We speculate that the longitudinal changes in HMW adiponectin levels observed in the women from the current study were associated with a gain in visceral and/or intramuscular fat mass rather than any changes in bodyweight.

Although the cause of the longitudinal reduction in female HMW adiponectin levels observed in the current study is unclear, such reduction might be associated with an increased risk of CVD in elderly post-menopausal women [22] because there was a low proportion of healthy elderly that were current smokers, the levels of non-HDL cholesterol remained unchanged, and adiponectin levels are associated with endothelial dysfunction [23]. Future studies are necessary to elucidate the clinical significance and mechanism by which HMW adiponectin levels were reduced in elderly women.

Recent studies have revealed the presence of an inverse relationship between the total adiponectin levels and muscle strength in Japanese females [24]. Such findings contradict our current findings, demonstrating that both HMW adiponectin levels and knee extension strength decreased at the 5-year follow-up in women. The discrepancy in the relationship between adiponectin levels and muscle strength might be due to the women in previous studies being younger than those in our study by as much as 20 years. Distinct mechanisms might affect the relationship between adiponectin levels and muscle strength during different stages of life.

Kizer et al. observed that the total adiponectin levels were inversely correlated with body weight in the univariate and multivariate analyses and demonstrated that the total adiponectin levels increased with age [5]. Although a reduction in physical ability with age was also observed in that study (consistent with the results of the current study), HMW adiponectin levels were decreased in the current study. Kizer et al. study observed that approximately one-third of the participants developed new difficulties with activities of daily living during the follow-up period; however, our study sample consisted of healthy elderly participants who lived independently. Therefore, this discrepancy might have been due to a difference in adiponectin levels, since the plasma adiponectin levels were positively correlated with the severity of frailty [25]. Therefore, changing adiponectin levels might be used to distinguish frail from the healthy elderly.

We also determined that the ADIPOQ SNPs included in the current study were not associated with the longitudinal HMW adiponectin levels. Most genetic studies to date have observed associations between certain ADIPOQ SNPs and the total adiponectin levels [4]. Here, we investigated the longitudinal association between HMW adiponectin concentrations and SNPs; however, we could not demonstrate any correlation between these two factors. Thus, in contrast to the total adiponectin levels, the longitudinal HMW adiponectin levels might be affected to a greater extent by pathophysiological and environmental determinants, (e.g., sex hormone levels [26] and diet [27]) than by genetic factors.

The present study had several limitations. First, we did not measure the total adiponectin levels. Therefore, we could not compare our findings regarding HMW adiponectin levels to those previously published on total adiponectin levels, nor could we examine the potential longitudinal association between the two forms of adiponectin. However, the total and HMW adiponectin have similar utilities in terms of assessing the adiponectin levels in the blood [28]. Therefore, the HMW adiponectin levels can act as a proxy for the total adiponectin levels. Second, we could not measure the fasting plasma glucose or glycosylated hemoglobin levels at baseline or follow-up. Therefore, we could not adjust our results for the presence of potential confounders, such as glucose intolerance or pre-diabetes. However, after reanalyzing the data following the exclusion of participants with diabetes mellitus at baseline, longitudinal HMW adiponectin levels remained significantly decreased in all participants and in only women. This finding indicates that the small percentage of participants with diabetes mellitus did not significantly affect the results. Third, information regarding cholesterol-lowering medication taken by the participants was not recorded; thus, the use of these medications may have confounded our findings. However, notwithstanding these limitations, our results which demonstrate HMW adiponectin levels significantly decreased longitudinally, despite being correlated with body weight and HDL cholesterol, are significant and novel.

## Conclusion

Therefore, the plasma HMW adiponectin levels were longitudinally and significantly decreased in elderly Japanese women. These findings may indicate an increased risk of CVD in elderly women. Further studies are necessary to determine the underlying mechanisms of age- and sex-related changes in adiponectin levels and their association with CVD and frailty in elderly populations.

## References

[1] Kissebah AH, Sonnenberg GE, Myklebust J (2000). Quantitative trait loci on chromosomes 3 and 17 influence phenotypes of the metabolic syndrome. Proc Natl Acad Sci USA.

[2] Weyer C, Funahashi T, Tanaka S (2001). Hypoadiponectinemia in obesity and type 2 diabetes: close association with insulin resistance and hyperinsulinemia. J Clin Endocrinol Metab.

[3] Dekker JM, Funahashi T, Nijpels G (2008). Prognostic value of adiponectin for cardiovascular disease and mortality. J Clin Endocrinol Metab.

[4] Hara K, Boutin P, Mori Y (2002). Genetic variation in the gene encoding adiponectin is associated with an increased risk of type 2 diabetes in the Japanese population. Diabetes.

[5] Kizer JR, Arnold AM, Strotmeyer ES (2010). Change in circulating adiponectin in advanced old age: determinants and impact on physical function and mortality. The Cardiovascular Health Study All Stars Study. J Gerontol A Biol Sci Med Sci.

[6] Cnop M, Havel PJ, Utzschneider KM (2003). Relationship of adiponectin to body fat distribution, insulin sensitivity and plasma lipoproteins: evidence for independent roles of age and sex. Diabetologia.

[7] Isobe T, Saitoh S, Takagi S (2005). Influence of gender, age and renal function on plasma adiponectin level: the Tanno and Sobetsu study. Eur J Endocrinol.

[8] Song HJ, Oh S, Quan S (2014). Gender differences in adiponectin levels and body composition in older adults: Hallym aging study. BMC Geriatr.

[9] Hozawa A, Sugawara Y, Tomata Y (2012). Relationship between serum adiponectin levels and disability-free survival among community-dwelling elderly individuals: the Tsurugaya project. J Gerontol A Biol Sci Med Sci.

[10] Oritsu M, Komatsu J, Hiyoshi T (2009). Fundamental evaluation of high molecular weight adiponectin by chemiluminescent enzyme immunoassay using Lumipulse and Lumipulse Presto II. Med Pharm.

[11] Sakurai T (1998). Fundamental study on measurement of total cholesterol by ultraviolet-end (UV-End) method using cholesterol dehydrogenase (CDH). Jpn J Med Technol.

[12] Nakai T, Ito K, Sekine K (1995). The basic study of Cholestest N HDL, HDL cholesterol measurement reagent, and the measurement of specific samples. Jpn J Clin Med.

[13] Nakayama T, Soma M, Rahmutula D (2001). Isolation of the 5′-flanking region of genes by thermal asymmetric interlaced polymerase chain reaction. Med Sci Monit.

[14] Sano M, Kuroi N, Nakayama T (2005). Association study of calcitonin-receptor-like receptor gene in essential hypertension. Am J Hypertens.

[15] Livak KJ, Marmaro J, Todd JA (1995). Towards fully automated genome-wide polymorphism screening. Nat Genet.

[16] Yamamoto Y, Hirose H, Saito I (2002). Correlation of the adipocyte-derived protein adiponectin with insulin resistance index and serum high-density lipoprotein-cholesterol, independent of body mass index, in the Japanese population. Clin Sci (Lond).

[17] Kato K, Osawa H, Ochi M (2008). Serum total and high molecular weight adiponectin levels are correlated with the severity of diabetic retinopathy and nephropathy. Clin Endocrinol (Oxf).

[18] Pajvani UB, Hawkins M, Combs TP (2004). Complex distribution, not absolute amount of adiponectin, correlates with thiazolidinedione-mediated improvement in insulin sensitivity. J Biol Chem.

[19] Janssen I, Heymsfield SB, Allison DB (2002). Body mass index and waist circumference independently contribute to the prediction of nonabdominal, abdominal subcutaneous, and visceral fat. Am J Clin Nutr.

[20] Goodpaster BH, Park SW, Harris TB (2006). The loss of skeletal muscle strength, mass, and quality in older adults: the health, aging and body composition study. J Gerontol A Biol Sci Med Sci.

[21] Kelly KR, Navaneethan SD, Solomon TP (2014). Lifestyle-induced decrease in fat mass improves adiponectin secretion in obese adults. Med Sci Sports Exerc.

[22] Kannel WB, Hjortland MC, McNamara PM (1976). Menopaused and risk of cardiovascular disease: the Framingham study. Ann Internal Med.

[23] Shimabukuro M, Higa N, Asahi T (2003). Hypoadiponectinemia is closely linked to endothelial dysfunction in man. J Clin Endocrinol Metab.

[24] Huang C, Niu K, Momma H (2014). Inverse association between circulating adiponectin levels and skeletal muscle strength in Japanese men and women. Nutr Metab Cardiovasc Dis.

[25] Tsai JS, Wu CH, Chen SC (2013). Plasma adiponectin levels correlate positively with an increasing number of components of frailty in male elders. PLoS One.

[26] Miyatani Y, Yasui T, Uemura H (2008). Associations of circulating adiponectin with estradiol and monocyte chemotactic protein-1 in postmenopausal women. Menopause.

[27] Chen JH, Ouyang C, Ding Q (2015). A moderate low-carbohydrate low-calorie diet improves lipid profile, insulin sensitivity and adiponectin expression in rats. Nutrients.

[28] Van Andel M, Drent ML, Van Herwaarden AE et al (2017) A method comparison of total and HMW adiponectin: HMW/total adiponectin ratio varies versus total adiponectin, independent of clinical condition. Clin Chim Acta 465:30–33 10.1016/j.cca.2016.12.009 27986548

